# A Case of Heparin-Induced Thrombocytopenia That Developed in the Therapeutic Course of Anti-Neutrophil Cytoplasmic Antibody-Associated Vasculitis

**DOI:** 10.1155/2019/2724304

**Published:** 2019-07-22

**Authors:** Taketoshi Nonaka, Makoto Harada, Masahiko Sumi, Wataru Ishii, Tohru Ichikawa, Mamoru Kobayashi

**Affiliations:** ^1^Department of Nephrology, Nagano Red Cross Hospital, 5-22-1 Wakasato, Nagano 380-8582, Japan; ^2^Department of Hematology, Nagano Red Cross Hospital, 5-22-1 Wakasato, Nagano 380-8582, Japan; ^3^Department of Rheumatology, Nagano Red Cross Hospital, 5-22-1 Wakasato, Nagano 380-8582, Japan

## Abstract

**Background:**

Heparin-induced thrombocytopenia (HIT) causes thrombocytopenia via an immunological mechanism, resulting in severe organ injury due to arterial-venous thrombosis. HIT often develops in hemodialysis patients owing to heparin use. Anti-neutrophil cytoplasmic antibody-associated vasculitis (AAV) is a systemic vasculitis, and cases of AAV complicated with HIT are rare. In addition, it mostly occurs in patients undergoing hemodialysis.

**Case Presentation:**

An 87-year-old woman presented with rapidly progressive renal failure and severe leg edema. She was diagnosed with AAV and treated with glucocorticoid and heparin calcium to prevent deep vein thrombosis. Eight days after the start of heparin calcium, her platelet count decreased and the anti-platelet factor 4-heparin complex antibody was strongly positive (>5.0 U/mL; the cutoff point of the anti-platelet factor 4-heparin complex antibody evaluated by the latex turbidity assay is 1.0 U/mL). She was diagnosed with HIT and treated with argatroban. Subsequently, her platelet counts increased gradually.

**Conclusion:**

We encountered a case of HIT that developed prior to the induction of hemodialysis in the clinical course of AAV. When AAV clinical course presents thrombocytopenia, the possibility of HIT should be considered.

## 1. Introduction

Heparin-induced thrombocytopenia (HIT) causes thrombocytopenia and develops a severe arterial-venous thrombosis via the immunological mechanism, which produces antibodies targeting the platelet factor 4 (PF4) complex with heparin [1]. Argatroban is usually used as an initial therapy [1]. It has been reported that patients who undergo hemodialysis and patients with autoimmune disorders are significantly associated with developing HIT [2, 3]. The reasons are as follows: most patients undergoing hemodialysis are treated with heparin in the hemodialysis session, and autoimmune disorders may be the risk factor in the abnormal production of the antigen-antibody complex [2, 3].

Anti-neutrophil cytoplasmic antibody-associated vasculitis (AAV) is a systemic vasculitis characterized by the presence of anti-neutrophil cytoplasmic antibodies (ANCAs) [4]. AAV often causes severe organ injury such as alveolar hemorrhage, interstitial pneumonia, and a rapid progressive glomerulonephritis resulting in dialysis [4].

Cases of coexistence of AAV and HIT are rare, but several cases do exist [5–8]. Most of these coexistent cases developed HIT after initiating hemodialysis due to AAV-induced renal failure. However, herein, we present a case of HIT that developed prior to the induction of hemodialysis in the clinical course of AAV and report the details of the clinical course and discuss the association between AAV and HIT.

## 2. Case Presentation

An 87-year-old woman who presented with appetite loss and leg edema was admitted for evaluation. Blood examination revealed an inflammatory response (C-reactive protein level was 7.85 mg/dL), kidney dysfunction (blood urea nitrogen was 37.4 mg/dL, and the serum creatinine level was 2.25 mg/dL), and hypoalbuminemia. Urinary examination revealed severe proteinuria (7.05 g/gCr) and hematuria. In addition, the patient was positive for myeloperoxidase-ANCA (147 U/mL). The main laboratory data are presented in Table 1.

**Figure 1:**
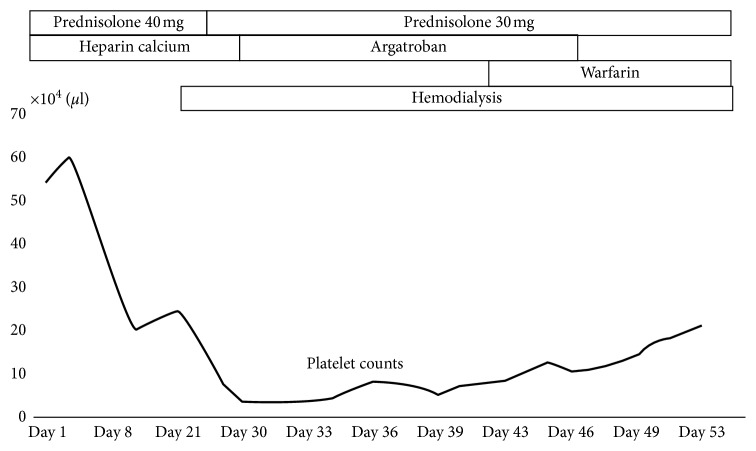
Clinical course of the platelet count in the current patient. Eight days after the start of heparin calcium, the platelet count gradually decreased.

According to these results, she was diagnosed with AAV and glucocorticoid therapy was started (an oral dose of prednisolone 40 mg/day). The patient was considered to be at a high risk of deep vein thrombosis; therefore, heparin calcium therapy was also administered. Although the systemic inflammation improved after glucocorticoid therapy, the leg edema and hypoalbuminemia did not improve. Her body weight steadily increased, and leg edema worsened 14 days after hospitalization; she received hemodialysis therapy. Although the baseline platelet count was 400,000 to 500,000/*μ*L, 8 days after the start of heparin calcium therapy, her platelet count gradually decreased. In addition, blood examination performed 26 days after hospitalization revealed that the platelet count was 81,000/*μ*L, the fibrinogen level was low at 138 mg/dL, and the FDP-D-dimer level was high at 18.2 *μ*g/mL (Figure 1). According to the 4Ts scoring, reduction of 50% or more of the platelet count and a platelet decrease between 5 and 10 days after using heparin are consistent. Additionally, because the FDP-D-dimer was high, she might have had thrombosis. Other causes of thrombocytopenia were not detected. Therefore, it was highly possible that she developed HIT. In addition, the patient was strongly positive for the anti-PF4-heparin complex antibody (the titer of the anti-platelet factor 4-heparin complex antibody evaluated by the latex turbidity assay was more than 5.0 U/mL). The titer measurement result was extremely high because the cutoff point of the anti-platelet factor 4-heparin complex antibody evaluated by the latex turbidity assay is 1.0 U/mL. These results were the basis of the HIT diagnosis. We performed Doppler echography for her legs to evaluate deep vein thrombosis 28 days after hospitalization. However, deep vein thrombosis was not detected. Because we expected her renal function to recover at that time, enhanced computed tomography to evaluate systemic thrombosis was not performed. Heparin was discontinued 29 days after hospitalization, and argatroban therapy was administered, following which warfarin therapy was administered. After starting argatroban therapy, her platelets count gradually increased (Figure 1). Fortunately, no symptoms due to arterial or venous thrombosis presented in her clinical course. Fifty-two days after her hospitalization, the platelet count improved to 200,000/*μ*L. Because her AAV activity was well controlled and the titer of MPO-ANCA was reduced, the dose of prednisolone was decreased. However, because kidney function was not recovered, she was administered maintenance hemodialysis therapy.

## 3. Discussion

In the current case, heparin therapy was administered because of a high risk for developing deep vein thrombosis and HIT developed prior to hemodialysis. Concerning the clinical course of the current case, 8 days after the start of heparin calcium therapy, her platelet count gradually decreased. This would be consistent with HIT. In addition, heparin was used in the hemodialysis session until 29 days after hospitalization. This is the reason the platelet count continued to decrease. In the current case, the titer of the anti-platelet factor 4-heparin complex antibody was extremely high at more than 5.0 U/mL (the cutoff point by the latex turbidity assay is 1.0 U/mL). It is thought that a high titer of the anti-platelet factor 4-heparin complex antibody is the reason why the recovery of the platelet count from argatroban therapy was slow.

Kato et al. have noted that hemodialysis, autoimmune diseases, gout, and heart failure were significantly correlated with HIT in their retrospective observational study. Most patients undergoing hemodialysis are treated with heparin in their dialysis session, and cases of HIT are often reported [2]. A previous study from Japan reported that the incidence of HIT in patients undergoing hemodialysis was 3.9% [2]. Kato et al. have performed a multivariate analysis of 55 cases of in-hospital HIT and concluded that there is a significant association between HIT and autoimmune diseases [3]. There are rare but several cases of HIT in the clinical course of AAV [5–8]. We present the clinical characteristics of previously published four coexisting cases of AAV and HIT (Table 2) [5–8].

The age range was 40 to 91 years; the ratio of MPO-to-proteinase 3 ANCA was 1 : 1, and male-to-female ratio was 3 : 1. These cases showed severe organ injury, a systemic severe kidney injury requiring hemodialysis and an acute lung injury due to alveolar hemorrhage, and bronchiolitis obliterans resulting in pneumonia. Four cases were treated with methylprednisolone pulse therapy (250 to 1,000 mg) and a high dose of prednisolone, two were treated with cyclophosphamide, and one was treated with plasma exchange therapy. All cases developed HIT after the start of these immunosuppressive therapies and hemodialysis. Even with strong immunosuppressive therapy, the production of antibodies targeting the PF4 complex with heparin may not be suppressed. The previous reports of treating HIT by immunosuppressive therapy are really limited. Although Schell et al. treated HIT by plasma exchange and rituximab, the patient required bilateral limb amputation [9]. Previous research using murine models has indicated that production of the anti-PF4-heparin complex antibody is regulated by not only B cells but also CD4 T cells [10]. Therefore, T-cell suppression may be also important to decrease the production of the anti-PF4-heparin complex antibody. Although all these cases developed HIT after the start of hemodialysis, the patient in our case developed HIT before the initiation of hemodialysis. There was no relationship between the HIT occurrence and hemodialysis in the current case. AAV may be associated with the development of HIT. In addition, patients with AAV are likely to be treated with heparin because patients with AAV often require plasma exchange therapy and/or hemodialysis and AAV is a high risk factor for deep vein thrombosis [1, 11]. Therefore, we should consider the possibility of HIT when patients with AAV develop thrombocytopenia. However, because coexisting cases of AAV and HIT are uncommon, further studies of these types of cases are required to determine the significant association between both diseases.

In conclusion, we should consider the possibility of HIT when patients with AAV develop thrombocytopenia after the start of heparin use.

## Figures and Tables

**Table 1:** Main clinical data of the current case at hospital admission.

Urinalysis	
Protein	2+
	7.08 g/gCr
Hematuria	3+
	10–19 HPF
Blood analysis	
WBC	9500 *μ*L
Neut	80%
Lym	11.6%
Mono	7.6%
Eos	0.6%
Baso	0.2%
Hb	7.2 g/dL
Plt	431,000 *μ*L
Total protein	4.3 g/dL
Albumin	1.3 g/dL
BUN	37.4 mg/dL
Cr	2.25 mg/dL
UA	6.1 mg/dL
Na	135 mEq/L
K	4.4 mEq/L
Cl	106 mEq/L
AST	15 U/L
ALT	4 U/L
LDH	157 U/L
ALP	153 U/L
*γ*GT	20 U/L
T. bil	0.4 mg/dL
RF	72 IU/mL
ANA (homogeneous)	×20
C3	98 mg/dL
C4	31 mg/dL
CH50	42.8 U/mL
MPO-ANCA	147 U/mL
PR3-ANCA	<1.0 U/mL
Anti-GBM antibody	<2.0 U/mL

WBC, white blood cells; Hb, hemoglobin; Plt, platelet; BUN, blood urea nitrogen; Cr, creatinine; UA, uric acid; Na, sodium; K, potassium; Cl, chloride; AST, aspartate aminotransferase; ALT, alanine aminotransferase; LDH, lactate dehydrogenase; ALP, alkaline phosphatase; *γ*GT, gamma-glutamyl transpeptidase; T. bil, total bilirubin; RF, rheumatoid factor; ANA, antinuclear antibody; C3, complement 3; C4, complement 4; CH50, complement hemolytic activity assay; MPO-ANCA, myeloperoxidase-anti-neutrophil cytoplasmic antibody; PR3-ANCA, proteinase 3-anti-neutrophil cytoplasmic antibody; anti-GBM antibody, anti-glomerular basement membrane antibody.

**Table 2:** Clinical characteristics of previously published four coexisting cases of anti-neutrophil cytoplasmic antibody-associated vasculitis and heparin-induced thrombocytopenia.

	Sex	Age	Type of ANCA	Type of organ injury	Treatment	Onset of HIT and type of heparin
Roe et al. [5]	M	65	PR3	Crescentic glomerulonephritis, pulmonary hemorrhage	mPSL pulse, PSL, CY, hemodialysis	9 days after the start of hemodialysis, unfractionated heparin
Kaneda et al. [6]	F	91	MPO	Kidney dysfunction, pulmonary hemorrhage	mPSL pulse, PSL, hemodialysis	13 days after the start of hemodialysis, unfractionated heparin
Mandai et al. [7]	M	40	MPO	Crescentic glomerulonephritis, interstitial pneumonia	mPSL pulse, PSL, CY, PE, hemodialysis	5 days after the start of hemodialysis, unfractionated heparin
Thong et al. [8]	M	71	PR3	Kidney dysfunction	mPSL pulse, PSL, CY, hemodialysis	15 days after the start of hemodialysis, unfractionated heparin and dalteparin

ANCA, anti-neutrophil cytoplasmic antibody; CY, cyclophosphamide therapy; F, female; HIT, heparin-induced thrombocytopenia; M, male; MPO, myeloperoxidase; mPSL, methylprednisolone; PE, plasma exchange therapy (including double filtration plasmapheresis); PR3, proteinase 3; PSL, prednisolone.
